# Generative AI in Medical Practice: In-Depth Exploration of Privacy and Security Challenges

**DOI:** 10.2196/53008

**Published:** 2024-03-08

**Authors:** Yan Chen, Pouyan Esmaeilzadeh

**Affiliations:** 1 Department of Information Systems and Business Analytics College of Business Florida International University Miami, FL United States

**Keywords:** artificial intelligence, AI, generative artificial intelligence, generative AI, medical practices, potential benefits, security and privacy threats

## Abstract

As advances in artificial intelligence (AI) continue to transform and revolutionize the field of medicine, understanding the potential uses of generative AI in health care becomes increasingly important. Generative AI, including models such as generative adversarial networks and large language models, shows promise in transforming medical diagnostics, research, treatment planning, and patient care. However, these data-intensive systems pose new threats to protected health information. This Viewpoint paper aims to explore various categories of generative AI in health care, including medical diagnostics, drug discovery, virtual health assistants, medical research, and clinical decision support, while identifying security and privacy threats within each phase of the life cycle of such systems (ie, data collection, model development, and implementation phases). The objectives of this study were to analyze the current state of generative AI in health care, identify opportunities and privacy and security challenges posed by integrating these technologies into existing health care infrastructure, and propose strategies for mitigating security and privacy risks. This study highlights the importance of addressing the security and privacy threats associated with generative AI in health care to ensure the safe and effective use of these systems. The findings of this study can inform the development of future generative AI systems in health care and help health care organizations better understand the potential benefits and risks associated with these systems. By examining the use cases and benefits of generative AI across diverse domains within health care, this paper contributes to theoretical discussions surrounding AI ethics, security vulnerabilities, and data privacy regulations. In addition, this study provides practical insights for stakeholders looking to adopt generative AI solutions within their organizations.

## Introduction

Artificial intelligence (AI) is transforming many industries, including health care. AI has the potential to revolutionize health care by enabling the detection of signs, patterns, diseases, anomalies, and risks. From administrative automation to clinical decision support, AI holds immense potential to improve patient outcomes, lower costs, and accelerate medical discoveries [1]. An especially promising subset of AI is generative models, which are algorithms that can synthesize new data, imagery, text, and other content with humanlike creativity and nuance based on patterns learned from existing data [2]. Generative AI could power clinical practices in health care, from generating synthetic patient data to augmenting rare disease research to creating AI-assisted drug discovery systems [3]. Generative AI has the potential to detect signs, patterns, diseases, anomalies, and risks and assist in screening patients for various chronic diseases, making more accurate and data-driven diagnoses and improving clinical decision-making [4]. Generative AI also has the potential to transform patient care with generative AI virtual health assistants [5].

However, generative AI systems pose acute privacy and security risks along with their transformative potential because of their vast data requirements and opacity [6]. Generative AI models can be trained on sensitive, multimodal patient data, which could be exploited by malicious actors. Therefore, the collection and processing of sensitive patient data, along with tasks such as model training, model building, and implementing generative AI systems, present potential security and privacy risks. Given the sensitive nature of medical data, any compromise can have dire consequences, not just in data breaches but also in patients’ trust and the perceived reliability of medical institutions. As these AI systems move from laboratory to clinical deployment, a measured approach is required to map and mitigate their vulnerabilities. Another challenge of using generative AI models is that they can be biased, which could lead to inaccurate diagnoses and treatments [7].

Despite the growing interest in generative AI in health care, there is a gap in the literature regarding a comprehensive examination of the unique security and privacy threats associated with generative AI systems. Our study attempts to provide insights into the different categories of generative AI in health care, including medical diagnostics, drug discovery, virtual health assistants, medical research, and clinical decision support. This study also aims to address the gap by identifying security and privacy threats and mapping them to the life cycle of various generative AI systems in health care, from data collection through model building to clinical implementation. By identifying and analyzing these threats, we can gain insights into the vulnerabilities and risks associated with the use of generative AI in health care. We also seek to contribute to theory and practice by highlighting the importance of addressing these threats and proposing mitigation strategies.

The findings of this study can inform the development of future generative AI systems in health care and help health care organizations better understand the potential benefits and risks of using these systems. The significance of this study lies in its potential to inform policy makers, health care organizations, and AI developers about the security and privacy challenges associated with generative AI in health care. The findings of this study can guide the development of robust data governance frameworks, secure infrastructure, and ethical guidelines to ensure the safe and responsible use of generative AI in health care. With careful governance, the benefits of generative models can be realized while safeguarding patient data and public trust. Ultimately, this study contributes to the advancement of knowledge in the field of AI in health care and supports the development of secure and privacy-preserving generative AI systems for improved patient care and outcomes.

## Generative AI Applications in Health Care

### Overview

Generative AI models use neural networks to identify patterns and structures within existing data to generate new and original content. Generative AI refers to techniques such as generative adversarial networks (GANs) and large language models (LLMs) that synthesize novel outputs such as images, text, and molecular structures [8]. GANs use 2 neural networks, a generator and a discriminator, that compete against each other to become better at generating synthetic data [9]. LLMs such as GPT-4 (OpenAI) are trained on massive text data and can generate synthetic natural language text, code, and so on [10].

Generative AI has spurred a wide range of applications in health care. This subset of AI has the potential to make a breakthrough in medical diagnostic applications, given its capability to build models using multimodal medical data [5]. Generative AI also promises to accelerate drug discovery by inventing optimized molecular candidates [11]. In research settings, these generative AI techniques can hypothesize promising new directions by creatively combining concepts [12]. Generative AI also has applications in engaging patients through natural conversation powered by LLMs [2]. When integrated into clinical workflows, it may also provide physicians with patient-specific treatment suggestions [13].

The classification of generative AI systems presented in Table 1 was developed based on a careful analysis of the various factors that differentiate these technologies.

**Table 1:** Categories of generative artificial intelligence (AI) applications in health care.

Category	Example	Setting	User	Input data	Output data	Personalization level	Workflow integration	Validation needed	Impact	Risks	Human involvement
Medical diagnostics	AI-Rad Companion	Radiology	Radiologists	Medical images	Text findings	Individual	Postimaging	High	Improved diagnosis	Reliability and bias	High
Drug discovery	Insilico Medicine	Biotechnology	Research scientists	Target proteins and disease data	Novel molecular structures	Semipersonalized	Early-stage research	High	Faster discoveries	Safety and testing requirements	Moderate
Virtual health assistants	Sensely	Web clinics	Patients	Conversation	Conversation	Semipersonalized	Patient engagement	Moderate	Increased access	Privacy and misinformation	Moderate
Medical research	Anthropic	Laboratories and academia	Researchers	Research concepts and data sets	Hypotheses and questions	Semipersonalized	Idea generation	Low	Research insights	Misdirection	Moderate
Clinical decision support	Glass AI	Point of care	Physicians	Patient data	Treatment suggestions	Individual	Diagnosis and treatment	High	Improved outcomes	Overreliance and bias	High

### Differentiating Factors

The goal was to provide a framework for better understanding the diversity of generative AI across health care settings. We leverage several key factors to differentiate the applications and provide insights into this emerging field, described in the following sections.

#### Setting

The clinical setting categorizes where in the health care workflow the generative AI system is applied, such as diagnostics, treatment planning, drug discovery, clinical decision support, and patient education [14]. This provides insights into the breadth of health care contexts leveraging these technologies.

#### Users

Generative AI tools are tailored to different types of users in health care, from clinicians to researchers to patients [15]. Categorization by intended user groups reveals how generative AI penetrates various stakeholder groups and which user groups may adopt and interact with generative AI applications.

#### Input Data

The data sources powering generative AI systems vary significantly, from electronic health records (EHRs) and medical imaging to biomedical literature, laboratory tests, and patient-provided data [16]. Categorization by data inputs illustrates how different data fuel different categories of applications.

#### Output Data

The outputs produced by the system, such as images, care planning, prescription advice, treatment options, drug molecules, text, risk scores, and education materials [17], demonstrate the wide range of generative AI capabilities in health care.

#### Personalization Level

The level of personalization to individual patients reveals the precision of the outputs, from generalized to fully patient specific. This provides a perspective on the customizability of the generative AI system.

#### Workflow Integration

Some generative AI systems are designed as stand-alone applications, whereas others are integrated into clinical workflows via EHRs, order sets, and so on. Categorization by workflow integration sheds light on the level of adoption, implementation practices, and integration of these tools.

#### Validation Needs

The extent of validation required, from noncritical outputs to those needing rigorous US Food and Drug Administration approval [18], highlights differences in oversight and impact levels.

Impact: profiling the benefits and use cases served by the generative AI technology, such as improving diagnostics, reducing medication errors, or accelerating drug discovery, provides insights into the varied impacts.

#### Risks

Discussing risks and limitations provides a balanced view of concerns such as algorithmic bias, privacy concerns, security issues, system vulnerability, and clinical integration challenges.

#### Human-AI Collaboration

Generative AI systems differ in the level of human involvement required, from fully automated to human-in-the-loop (human engagement in overseeing and interacting with the AI’s operational process) [19]. Categorization by human-AI partnership provides insights into the changing dynamics between humans and AI across health care.

### Aims

This study aims to reveal crucial differences, use cases, adoption levels, various risks, and implementation practices by developing categories based on these key attributes of generative AI systems. The proposed framework clarifies the heterogeneous landscape of generative AI in health care and enables a trend analysis across categories. These factors provide a perspective on how generative AI manifests distinctly for various users, data types, workflows, risk factors, and human-AI partnerships within health care. By systematically analyzing the diverse range of generative AI systems across health care settings using the key factors discussed previously, we can classify the heterogeneous landscape of generative AI in health care into 5 overarching categories: medical diagnostics, drug discovery, virtual health assistants, medical research, and clinical decision support.

### Medical Diagnostics

Generative AI techniques can analyze data from wearables, EHRs, and medical images (eg, x-rays, magnetic resonance imaging, and computed tomography scans) to detect signs, patterns, diseases, anomalies, and risks and generate descriptive findings to improve diagnoses. Systems such as AI-Rad Companion leverage natural language generation models to compose radiology reports automatically, highlighting potential abnormalities and issues for clinician review [20]. This assists radiologists by providing initial draft findings more rapidly. However, clinicians must thoroughly validate any generative AI outputs before clinical use. Ongoing challenges include reducing false positives and negatives [21].

### Drug Discovery

Generative AI shows promise for expediting and enhancing drug discovery through inventing optimized molecular structures de novo. Techniques such as GANs combined with reinforcement learning allow the intelligent generation of molecular graph representations [22]. Companies such as Insilico Medicine are using these generative chemistry techniques to propose novel target-specific drug candidates with desired properties. This accelerates preclinical pharmaceutical research. However, validating toxicity and efficacy remains critical before human trials.

### Virtual Health Assistants

Generative models such as LLMs can power conversational agents that understand and respond to patient questions and concerns [23]. Companies such as Sensely and Woebot Health leverage these techniques to create virtual assistants that explain symptoms, provide health information, and offer screening triage advice through natural dialogue [24]. This increases access and engagement for patients. However, challenges remain around privacy, information accuracy, and integration into provider workflows [25].

### Medical Research

In research settings, generative AI can formulate novel hypotheses by making unexpected combinations of concepts, mimicking human creativity and intuition. Claude from Anthropic can read research papers and propose unexplored directions worth investigating [26]. This unique generative capacity could accelerate scientific advancement. However, corroboration by human researchers is crucial to prevent the blind acceptance of AI-generated findings [27].

### Clinical Decision Support

Integrating generative AI into clinical workflows could provide patient-specific suggestions to assist physicians in decision-making. Glass AI leverages LLMs such as GPT-3 to generate tailored treatment options based on patient data for physicians to review [15]. This could improve outcomes and reduce errors. However, bias mitigation and high validation thresholds are critical before real-world adoption [28].

By holistically examining all the key factors, we can see how each one contributes to delineating these 5 high-level categories that provide a comprehensive snapshot of the generative AI landscape in health care. Analyzing these 5 categories through the lens of the proposed factors enables our study to reveal crucial differences, use cases, benefits, limitations, and implementation practices of generative AI technologies across major health care domains.

## Literature Review

The adoption of AI (powered by various models) is accelerating across health care for applications ranging from medical imaging to virtual assistants. However, the data-intensive nature and complexity of these systems introduce acute privacy and security vulnerabilities that must be addressed to ensure safe and ethical deployment in clinical settings. This literature review covers 2 topics. First, we highlight the dual nature of technological advancements in generative AI within health care, its benefits, and its risks, particularly in terms of privacy and security that it entails. Second, we explain AI regulation and compare the key aspects of the European Union (EU) AI Act and the US AI Bill of Rights.

## Generative AI: Balancing Benefits and Risks

### Overview

The use of generative AI systems in medicine holds promise for improvements in areas such as patient education and diagnosis support. However, recent studies highlight that privacy and security concerns may slow user adoption. A survey explores the application of GANs toward ensuring privacy and security [29]. It highlights how GANs can be used to address increasing privacy concerns and strengthen privacy regulations in various applications, including medical image analysis. The unique feature of GANs in this context is their adversarial training characteristic, which allows them to investigate privacy and security issues without predetermined assumptions about opponents’ capabilities. This is crucial because these capabilities are often complex to determine with traditional attack and defense mechanisms. In the privacy and security models using GANs, the generator can be modeled in two ways: (1) as an attacker aiming to fool a defender (the discriminator) to simulate an attack scenario and (2) as a defender resisting a powerful attacker (the discriminator) to simulate a defense scenario.

Examples of defense models include generative adversarial privacy [30], privacy-preserving adversarial networks [31], compressive adversarial privacy [32], and reconstructive adversarial network [33]. These GAN-based mechanisms offer innovative ways to enhance privacy and security in various machine learning and data processing scenarios. The examples are described in the subsequent sections.

### Protection of Preimage Privacy

The compressive privacy GAN is designed to preprocess private data before the training stage in machine learning as a service scenarios [34]. It includes 3 modules: a generator module (G) as a privatization mechanism for generating privacy-preserving data, a service module (S) providing prediction services, and an attacker module (A) that mimics an attacker aiming to reconstruct the data. The objective is to ensure optimal performance of the prediction service, even in the face of strong attackers, by intentionally increasing the reconstruction error. This method defends against preimage privacy attacks in machine learning as a service by ensuring that the input data of a service module contains no sensitive information.

### Privacy in Distributed Learning Systems

In decentralized learning systems, such as distributed selective stochastic gradient descent [35] and federated learning (FL) [36], data are trained locally by different participants without data sharing. This setup can protect data privacy to some extent, but it is not perfect. The GAN-based models in these systems can mimic data distribution and potentially threaten data privacy. The potential risks associated with the application of GAN-based models in decentralized learning systems are multifaceted, highlighting the need for robust privacy protection measures. These risks are explained as the following: an attacker might use GANs to recover sensitive information within the distributed training system, and a malicious server can reveal user-level privacy in distributed learning systems by training a multitask GAN with auxiliary identification.

Protection mechanisms include embedding a “buried point layer” in local models to detect abnormal changes and block attackers and integrating GAN with FL to produce realistic data without privacy leakage.

### Differential Privacy in GANs

To address the problem of privacy leakage in the models, two solutions have been proposed: (1) adding a regularization term in a loss function to avoid overfitting and improve robustness; for example, this method can be applied to defend against membership inference attacks, [37] and (2) adding acceptable noise into the model parameters to hinder privacy inference attacks. Such methods have been used for privacy protection, particularly the combination of differential privacy and neural networks [38].

In medical research, the widespread use of medical data, particularly in image analysis, raises significant concerns about the potential exposure of individual identities. An innovative adversarial training method focused on identity-obfuscated segmentation has been proposed to address this challenge [39]. This method is underpinned by a deep convolutional GAN-based framework comprising three key components: (1) a deep encoder network, functioning as the generator, efficiently obscuring identity markers in medical images by incorporating additional noise; (2) a binary classifier serves as the discriminator, ensuring that the transformed images retain a resemblance to their original counterparts; and (3) a convolutional neural network–based network dedicated to medical image analysis, acting as an alternate discriminator responsible for analyzing the segmentation details of the images. This framework integrates an encoder, a binary classifier, and a segmentation analysis network to form a robust approach to safeguard medical data privacy while preserving the integrity and efficacy of medical image segmentation.

The use of EHR medical records has significantly advanced medical research while simultaneously amplifying concerns regarding the privacy of this sensitive information. In response, Choi et al [40] devised the medical GAN (medGAN), an innovative adaptation of the standard GAN framework, aimed at producing synthetic patient records that respect privacy. The medGAN excels at generating high-dimensional discrete variables. Its architecture uses an autoencoder as the generator, which creates synthetic medical data augmented with noise. A binary classifier functions as the discriminator, ensuring the resemblance of these data to real records. The outcome is synthetic medical data suitable for various uses, such as distribution analysis, predictive modeling, and medical expert evaluations, minimizing the privacy risks associated with both identity and attributes. Furthering these advancements, Yale et al [41] conducted an in-depth evaluation of medGAN’s ability to protect privacy in medical records. In a parallel development, Torfi and Fox [42] introduced Correlation-Capturing Convolutional Generative Adversarial Networks (CorGAN), which focuses on the correlations within medical records. Unlike medGAN, CorGAN uses a dual autoencoder in its generator, enabling the creation of sequential EHRs rather than discrete entries. This approach enhances predictive accuracy, providing more effective assistance to medical professionals [43].

Similarly, Nova [14] discusses the transformative impact of generative AI on EHRs and medical language processing, underlining the accompanying privacy concerns. It examines the balance between the utility of GANs in generating health care data and the preservation of privacy. Rane [44] explores the wider privacy and security implications of using generative AI models, such as ChatGPT, in health care within the context of Industry 4.0 and Industry 5.0 transformation. The impact of generative content on individual privacy is further explored by Bale et al [45], emphasizing the ethical considerations in health care.

Ghosheh et al [46] suggest that the use of GANs to create synthetic EHRs creates many privacy challenges (eg, reidentification and membership attacks). Hernandez et al [47] discuss privacy concerns related to synthetic tabular data generation in health care. Various methods and evaluation metrics are used to assess the privacy dimension of the synthetic tabular data generation approaches. These methods include identity disclosure, attribute disclosure, distance to the closest record, membership attack, maximum real-to-synthetic similarity, differential privacy cost, and GANs. For instance, differential privacy is an approach that adds noise to the data to prevent the identification of individuals. GANs can create new and nonreal data points. Other advanced statistical and machine learning techniques attempt to balance data utility and privacy. Each method has its strengths and limitations, and the choice depends on the specific requirements of the health care application and the sensitivity of the data involved.

The applications and challenges of generative AI in health care, including privacy issues and AI-human collaboration, are explored by Fui-Hoon et al [48]. They discuss several privacy issues related to generative AI, such as the potential disclosure of sensitive or private information by generative AI systems, the widening of the digital divide, and the collection of personal and organizational data by these systems, which raises concerns about security and confidentiality. In addition, they highlight regulatory and policy challenges, such as issues with copyright for AI-generated content, the lack of human control over AI behavior, data fragmentation, and information asymmetries between technology giants and regulatory authorities.

A study discusses the potential of FL as a privacy-preserving approach in health care AI applications [49]. FL is a distributed AI paradigm that offers privacy preservation in smart health care systems by allowing models to be trained without accessing the local data of participants. It provides privacy to end users by only sharing gradients during training. The target of FL in health care AI applications is to preserve the privacy of sensitive patient information communicated between hospitals and end users, particularly through Internet of Medical Things (IoMT) devices. The approach incorporates advanced techniques such as reinforcement learning, digital twin, and GANs to detect and prevent privacy threats in IoMT networks. The potential beneficiaries of FL in health care include patients, health care providers, and organizations involved in collaborative health care research and analysis. However, implementing FL in IoMT networks presents challenges, such as the need for robust FL for diffused health data sets, the integration of FL with next-generation IoMT networks, and the use of blockchain for decentralized and secure data storage. Furthermore, incentive mechanisms are being explored to encourage the participation of IoMT devices in FL, and digital twin technology is being leveraged to create secure web-based environments for remote patient monitoring and health care research. Overall, FL in health care AI applications aims to address privacy and security concerns while enabling collaborative and efficient health care systems.

Another study emphasizes the need for secure and robust machine learning techniques in health care, particularly focusing on privacy and security [50]. Finally, a study addresses the vulnerabilities of generative models to adversarial attacks (eg, evasion attacks and membership inference attacks), highlighting a significant area of concern in health care data security [51]. These studies collectively underscore the need for a balanced approach to leveraging the benefits of AI-driven health care innovations while ensuring robust privacy and security measures.

## AI, Legal Challenges, and Regulation

AI, especially generative AI, has presented many legal challenges, raising many profound questions on how AI can be legally, securely, and safely used by businesses and individuals [52]. The EU AI Act, passed in 2023, is the first comprehensive legal framework to specifically regulate AI systems [53]. It categorizes systems by risk level and introduces mandatory requirements for high-risk AI related to data and documentation, transparency, human oversight, accuracy, cybersecurity, and so on. As stated in the act, national authorities will oversee compliance.

The US AI Bill of Rights, unveiled in 2023, takes a different approach as a nonbinding set of principles to guide AI development and use focused on concepts such as algorithmic discrimination awareness, data privacy, notice and explanation of AI, and human alternatives and oversight [54]. Rather than authoritative regulation, it promotes voluntary adoption by organizations.

Although the EU law institutes enforceable accountability around risky AI, the US bill espouses aspirational AI ethics principles. Both identify important issues such as potential bias, privacy risks, and the need for human control but tackle them differently—the EU through compliance requirements and the United States through voluntary principles. Each seeks more responsible AI but via divergent methods that fit their governance models. Despite differences in methods, there is a consensus on fundamental issues such as ensuring transparency, maintaining accuracy, minimizing adverse effects, and providing mechanisms for redressal.

Specifically, for generative AI such as ChatGPT, the EU AI Act mandates transparency requirements, such as disclosing AI-generated content, designing models to prevent illegal content generation, and publishing training data summaries. Although the principles mentioned in the US AI Bill of Rights do not specifically address generative AI, they provide a framework for the ethical and responsible use of all AI technologies, including generative AI. The principles emphasize safety, nondiscrimination, privacy, transparency, and human oversight, all of which are relevant to developing and deploying generative AI systems.

Ultimately, the EU legislates binding rules that companies must follow, whereas the United States issues guidance that organizations may freely adopt. Despite this schism, both highlight growing policy makers’ concern over AI’s societal impacts and the emergence of either compulsory or optional frameworks aimed at accountability. As leading AI powers craft different but related policy solutions, ongoing collaboration around shared values while allowing varied implementations will be important for setting global AI standards.

## Security and Privacy Threats in the Life Cycle of a Generative AI in Health Care System

### Overview

Although generative AI in health care holds great promise, substantial validation is required before real-world deployment. Ethical risks around reliability, accountability, algorithmic bias, and data privacy as well as security risks related to confidentiality, integrity, and availability must be addressed through a human-centric approach [55]. Liu et al [56] surveyed the security and privacy attacks related to machine learning and developed a taxonomy. The taxonomy classifies those attacks into three categories: (1) attacks targeting classifiers; (2) attacks violating integrity, availability, and privacy (ie, part of confidentiality); and (3) attacks with or without specificity. They also summarize the defense techniques in the training phase and the testing and inferring phase of the life cycle of machine learning, for example, data sanitization techniques against data poisoning attacks in the training phase and privacy-preserving techniques against privacy attacks in the testing or inferring phase. Similarly, Hu et al [57] present an overall framework of attacks and defense strategies based on the following five phases of the AI life cycle: (1) data collection phase—main security threats include databases, fake data, data breaches, and sensor attacks; defense strategies include data sanitization and data government; (2) data processing phase—image scaling is the main threat; recommended defense strategies include image reconstruction and data randomization; (3) training phase—data poisoning is the main threat; defense strategies focus on techniques that can identify and remove poisoned data (eg, the certified defense technique proposed by Tang et al [58]) and provide robust and reliable AI models; (4) inference phase—this phase mainly faces adversarial example attacks such as white-box, gray-box, and black-box attacks depending on how much the attacker knows about the target model; a variety of defense strategies can be implemented to tackle such attacks, such as adopting strategies in phases 1 to 3 to modify data (eg, data reconstruction and randomization) or modify or enhance models with newer model construction methods resistant to adversarial example attacks (eg, using deep neural networks and GAN-based networks [58,59]); (5) integration phase—AI models face AI biases, confidentiality attacks (eg, model inversion, model extraction, and various privacy attacks), and code vulnerability exploitation; defense strategies in this phase should be comprehensive via integrating various solutions such as fuzz testing and blockchain-based privacy protection.

Generative AI is built upon machine learning and AI techniques and hence faces similar security and privacy threats, as summarized in the studies by Liu et al [56] and Hu et al [57]. Nevertheless, because generative AI, such as LLMs, often requires large volumes of data (eg, large volumes of patient data) to train, it faces many existing and new security and privacy threats. If deployed carelessly, generative models increase the avenues for protected health information (PHI) to be leaked, stolen, or exposed in a breach. For example, deidentifying data for LLMs is challenging [60]. Even anonymized patterns in data could potentially reidentify individuals if models are improperly handled after training. One example is medical image analysis, as deidentified medical images could be reidentified in medical image analysis because of the massive amount of image data used in training [39]. LLMs in health care also face data quality and bias issues, similar to any machine learning model, leading to erroneous medical conclusions or recommendations [61].

**Figure 1:**
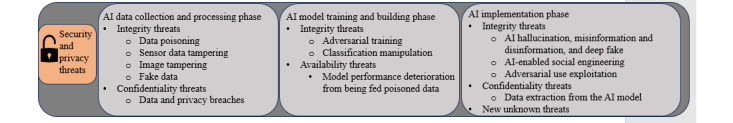
Artificial intelligence (AI) security and privacy threats in 3 phases of the AI life cycle.

Furthermore, hackers could also exploit vulnerabilities in systems hosting generative models to access the sensitive health data used for training. Skilled hackers may be able to feed prompts to models to obtain outputs of specific patient details that allow reidentification even from anonymized data. For example, improperly secured LLMs could enable bad actors to generate fake patient data or insurance claims [62]. In general, generative AI in health care encounters many of the same security and privacy threats as general AI and machine learning systems, along with new threats stemming from its unique context. On the basis of the life cycle in the studies by Liu et al [56] and Hu et al [57], our study presents a 3-phase life cycle for generative AI. It also identifies security and privacy threats and maps them to the life cycle of various generative AI systems in health care (Figure 1). It should be noted that although this study primarily discusses various security and privacy threats associated with generative AI in health care (such as AI hallucination in health care), many of these threats are not unique to generative AI systems and are also prevalent in broader AI systems and machine learning models in health care and other fields.

### Data Collection and Processing Phase

Similar to AI systems in other fields, almost all types of generative AI in health care face integrity threats. The main integrity threats in this phase are traditionally owing to errors and biases. Unintentionally, the increased data volume and complexity of generative AI threatens data integrity because errors and biases are prone to occur [63]. Errors and biases also depend on the data sources for different types of generative AI in health care. For example, assembling genomic databases and chemical compound or protein structure databases for drug discovery is extremely challenging and could be error ridden because many genomic and protein databases lack necessary annotations, are inconsistent in formats, and may be poor in data quality [64].

Intentionally, data poisoning can occur when data are collected from various software packages by tampering with data. For example, malicious insiders can tamper with data intentionally when gathering data from various software sources. For example, malicious actors can internationally submit mislabeled genomic sequences and chemical compound protein structures to tamper genomic databases and chemical compound or protein structure databases, leading to fault training models and AI hallucination.

In addition to data poisoning from software, in health care, data may be gathered from sensors embedded in medical devices and equipment. Sensor data can be spoofed [65,66], tampered with, and thus poisoned. Furthermore, medical data contains a large number of images. Adversaries can exploit the difference in cognitive processes between AI and humans and tamper with images during the data collection and processing phase. Image-scaling attacks, in which an adversary manipulates images so that changes are imperceptible to the human eye but recognizable by AI after downscaling, represent one such form of attack [67,68]. Other attacks on data sources of medical images include, but are not limited to, copy-move tampering (ie, copying an area and moving it to another area), classical inpainting tampering (ie, patching a missing area with tampered image slices), deep inpainting tampering (ie, similar to classical inpainting tampering but using highly realistic image slides generated by GANs), sharpening, blurring, and resampling [69]. In scenarios where AI in imaging diagnostics is targeted by such attacks, the image data can be poisoned with malicious information. Furthermore, generative AI, such as GANs, has empowered hackers to generate or change the attributes or content of medical images with high visual realism, making the detection of tampered images extremely difficult [69].

Moreover, many generative AI applications in health care rely on LLMs and are trained on large amounts of internet data without being properly screened and filtered [70]. Adversaries can use AI technologies to automatically generate large quantities of fake data to poison data to be fed into LLMs, resulting in deteriorated performance of the models (eg, accuracy and fairness) and eventually AI hallucination, misinformation or disinformation, and deepfakes. Although some of these threats are not unique to generative AI in health care, they can be particularly risky if false information is used for medical decision-making. Generative AI also carries unique integrity risks. As mentioned before, its capability to create synthetic data leads to a unique integrity risk—AI hallucination. In the health care context, generative AI in health care could be used to create fake medical records or alter existing ones. Fabricated medical data can be fed again into LLMs, further threatening the integrity of medical information. For instance, the malicious use of deepfakes generated by deep generative models could fabricate a patient’s medical history to falsely claim insurance or lead to incorrect treatments. Another example is that a generative AI model may create synthetic radiology reports to diagnose nonexistent medical conditions, leading to misdiagnosis or unnecessary treatment.

By contrast, research has used synthetic data in AI for medicine and health care to address the scarcity of annotated medical data in the real world [71]. For instance, deep generative models are used to create synthetic images such as skin lesions, pathology slides, colon mucosa, and chest x-rays, thereby greatly improving the reproducibility of medical data [71]. With the development of generative AI, researchers have increasingly used GANs to synthesize realistic training data for data imputation when the data lacks distribution. Noise-to-image and image-to-image GANs have been used to synthesize realistic training magnetic resonance imaging images to boost the performance of convolutional neural networks for image diagnostic AI [39,72]. CorGAN [42] synthesizes discrete and continuous health care records for model training. From a broader perspective, generative AI is projected to build and use next-generation synthetic gene networks for various AI applications in health care, including medical diagnostics, drug discovery, and medical research [73]. The growth in the use of synthetic data by generative AI also creates new concerns about data integrity and AI hallucination. Nevertheless, given that health care is a heavily regulated field in terms of patient privacy and safety, researchers even claim that synthetic medical data might be promising to overcome data sharing obstacles for health care AI and free developers from sensitive patient information [74]. These applications indicate that there is a fine line between harmful AI hallucinations or deepfakes and beneficial synthetic data use by generative AI in health care. Nevertheless, even the benevolent use of synthetic medical data faces privacy and security challenges as well as integrity challenges. Deep-faked patient face images could violate patient privacy and lead to the leakage or exploitation of PHI [75]. How to navigate this fine line is both a policy and research blind spot. Currently, there are just insufficient use cases, especially for rare use cases, to establish clinical reference standards such as clinical quality measures and evaluation metrics to assess risks and benefits.

Similar to generative AI applications in other fields, almost all types of generative AI in health care face confidentiality threats. Deidentified data may become identifiable during the data collection and processing phase, and confidential proprietary medical information, such as drug development and treatment plans, may be inferred during the data collection and processing phase [76], leading to data and privacy breaches. Research has found that genomic databases are prone to privacy violations. For example, legit researchers obtain or recover the whole or partial genomic sequence of a target individual (privacy violation through reference), link the sequence to a target individual (ie, reidentifying), and identify the group of interest of a target individual (privacy violation through membership reference) when processing data from multiple sources. In addition, the growth of synthetic medical data in health AI systems raises concerns about the vulnerabilities of such systems and the challenges of the current regulations and policies.

Table 2 summarizes the data sources and security or privacy threats for each type of generative AI in health care in the data collection and processing phase.

**Table 2:** Generative AI^a^ in health care categories, data sources, and security or privacy threats in the data collection and processing phase.

AI categories	Data source	Security and privacy threats	
		Unintentional (integrity and privacy threats)	Intentional (availability and integrity attacks)
Medical diagnostics	Medical images (eg, x-rays, CT^b^ scans, MRI^c^ scans, pet scans, and microscopy images) Patient reports and EHRs^d^ (eg, laboratory results, comorbidities, and symptoms) Clinical measurements (vital signs, tumor measurements, and fluid output) Patient metadata (demographics and family history) Expert annotations to train models	1-4: Incorrect, missing, or incomplete patient data or images occur owing to hardware or software errors, measurement and label errors, and human errors (eg, distorted images, partial images, and mismatched data or laboratory results or images) 1-4: Data integration errors occur when integrating data from various sources (eg, by mislabeling data attributes and mismatching patient information with their images and laboratory results) 1-4: Organic biases occur because of the nature of the disease and the demographics of patients, and selection biases rise because of human biases 5. Annotation errors and biases occur in all sources of data because of expert mistakes and human biases 1-4: Errors and bias in synthetic data or images 1-7: Privacy breaches (eg, reidentify patients)	1-3: Software tampering, medical sensor spoofing, medical equipment tampering or poisoning (eg, CT and MRI scanning equipment tampering), medical image tampering (eg, image scaling, copy-move tampering, sharpening, blurring, and resampling), generative fake data and images (eg, generative fake CT and MRI images undetectable by both human experts and generative AI), and medial data tampering or poisoning (eg, noise injection and maliciously synthesized data) 5: Annotation errors by intention
Drug discovery	Genomic databases (DNA or RNA sequencing data) Chemical compound or protein structure databases Bioactivity assay data (in vivo and in vitro) Disease or treatment knowledge bases (peer-reviewed findings) Patient clinical trial data Toxicity predictions from pharmacokinetic models	1-2: Duplication issues (eg, sequence redundancies or sequence duplications with minor variations), structural errors, and assembly or carried-over errors owing to poor data quality of sources 1-6: Data integration errors occur when integrating data from various sources 4-5: Wrong findings and errors in trials 1-6: Missing and incomplete data, missing or incorrect annotations, and human errors 1-6: Errors and bias in synthetic data 6: Incorrect or inaccurate models 1-7: Privacy breaches (eg, reidentify patients)	1-5: Genomic data tampering or poisoning (eg, maliciously forge and inject structures or sequences, analyses, and findings) 1-5: Annotation errors by intention 6: Model tampering
Virtual health assistants	EHRs Insurance claims data Patient symptom reports Mobile health data: data collected from mobile apps Speech and text inputs: data from patient interactions, including spoken dialogue and written communication Digitized medical reference information (guides and protocols) Custom health care knowledge bases	1-5: Incorrect, missing, or incomplete patient data 1-7: Data integration errors occur when integrating data from various sources 1-7: Organic biases occur because of the nature of the disease and the demographics of patients, and selection biases rise because of human biases 2: Errors owing to unknown fraudulent claims 6: Incorrect or inaccurate models 5-7: Errors and bias in synthetic data and AI hallucination 1-7: Privacy breaches (eg, reidentify patients)	1-7: Data or records tampering or poisoning (eg, noise injection using maliciously synthesized data, analyses, and findings) 1-7: Annotation errors by intention 1-7: AI hallucination
Medical research	Clinical trial and study data sets Epidemiological data from public health departments Biomedical publications and preprint archives Physician’s notes and patient diagnosis histories Genomics databases NIH^e^ open-source data repositories Biobanks: collections of biological samples	1-7: All the errors and biases mentioned in the above cells could be applicable	1-7: All the attacks mentioned in the above cells could be applicable
Clinical decision support	Real-time patient data feeds (vitals, laboratory results, etc) EHRs Population health data Hospital medical reference or treatment protocol guides Custom evidence-based clinical rules or guidelines Medical insurance claims data g. Pharmaceutical reference database	1-7: All the errors and biases mentioned in the above cells could be applicable	1-7: All the attacks mentioned in the above cells could be applicable

^a^AI: artificial intelligence.

^b^CT: computed tomography.

^c^MRI: magnetic resonance imaging.

^d^EHR: electronic health record.

^e^NIH: National Institutes of Health.

Again, it should be noted that although all AI and machine learning systems face many similar threats, as listed in Table 2, generative AI amplifies them because of its generating nature and data source volume and complexity. For example, generative medical research AI may update knowledge and literature databases with “wrong inputs” based on wrong findings in these databases or with synthesized but hallucinated findings. Similarly, generative virtual health assistants may put dangerous advice into knowledge databases based on erroneous data from sources or again put synthesized but hallucinated advice into such databases.

### Model Training and Building Phase

Generative AI also encounters integrity issues, leading to phenomena such as AI hallucinations during model training and development phases. This is especially true for generative AI in health care. Prior research found that generative AI created nonfactual or unfaithful data and outputs [72,77]. The growing use of highly synthetic data or images by generative AI, such as CorGAN, exacerbates the situation as it becomes increasingly challenging for human professionals to detect unfaithful data and outputs [69]. This can be a serious integrity and authenticity issue, as both patients and clinicians expect factual, scientific answers or outputs with consistency from such models. Technically speaking, similar to all other AI models, generative AI models in health care, particularly those based on deep learning, are often seen as “black boxes” [78]. The lack of interpretability and explainability can be a significant challenge in health care, where understanding the reasoning behind a diagnosis or treatment recommendation is crucial for integrity and accountability.

Adversarial training is a method to check for the integrity and accountability of AI models. The method uses carefully crafted adversarial examples to attack the training model to check for the integrity and robustness of outputs [57,79]. It is an active AI research area in the health care field. Adversarial training is used to check for fake or realistic features in synthetic medical images created by GANs to avoid fabrication and misleading in the model training process. By contrast, malicious parties also intensively explore this method and use adversarial examples to attack training models to generate incorrect outcomes [57]. Technically, all types of generative AI using GANs and LLMs, particularly those in health care, can be attacked with adversarial examples that compromise the integrity of the training model. For example, adversaries can use image-scaling attacks to feed human-invisible data into an AI model to force it to make a mistake [67,68].

Another example is to feed an AI model with carefully crafted relabeled data to create the wrong classification [80]. When being trained with adversarial examples, a diagnostic AI could make an incorrect diagnosis, a conversational virtual assistant could offer harmful advice to patients, and a clinical decision support AI could make the wrong recommendations, to list a few. Moreover, feeding an AI model with adversarial training examples and other poisonous data can also deteriorate the performance of AI, eventually making the AI model useless and thus unavailable. In general, adversarial attacks can pose long-term risks, such as thwarting AI innovation in health care because of concerns about misdiagnosis, mistreatment, and patient safety.

### Implementation Phase

In practice, generative AI systems in health care have been found to experiencing integrity threats, such as generating disinformation and misinformation, and making biased decisions [81]. AI hallucination is a newly-coined term describing the phenomenon wherein generative AI generates fake information that appears authentic [82]. If generative AI in health care is used for diagnostics, personalized medicine, or clinical assistance, AI hallucination can be extremely dangerous and may even harm patients’ lives [83]. As discussed before, because GANs and LLMs need large annotated medical data for training, the difficulty of acquiring such data (eg, unwillingness to share because of legal compliance requirements and data paucity resulting from rare medical conditions) leads to the proliferation of synthetic medical data creation. The relationship between AI hallucination by GANs and LLMs and synthetic data use is an unknown territory in research and practice, leading to unknown vulnerabilities such as adversarial attacks.

Privacy attacks are a grave concern at this stage. The use of GANs for creating synthetic EHRs and its associated privacy challenges are analyzed by Ghosheh et al [46]. Such privacy challenges are as follows: (1) risk of reidentification—although the data are synthetic, there might be a risk of reidentifying individuals if the synthetic data closely resemble real patient data; (2) data leakage—ensuring that the synthetic data do not leak sensitive information from the original data set; (3) model inversion attacks—potential for attackers to use the GAN model to infer sensitive information about the original data set. In this attack, attackers aim to reconstruct the training data using their ability to constantly query the model [84]; (4) membership inference attacks—an attacker gains access to a set of real patient records and tries to determine whether any of the real patients are included in the training set of the GAN model [85]; and (5) attribute disclosure attacks—an attacker can infer additional attributes about a patient by learning a subset of other attributes about the same patient [86].

Generative medical diagnosis and drug discovery AI involving genomic databases and chemical compound or protein structure databases are extremely susceptible to privacy attacks. Fernandes et al [87] pointed out that genomic data such as DNA data are susceptible to inference attacks, reidentification attacks, membership attacks, and recovery attacks. It is extremely concerning when such attacks target high-profile individuals. Moreover, generative AI enhances the ability to profile patients, thereby increasing the risk of privacy violations and attacks, although this capability is not unique to AI.

In addition to AI-specific security and privacy threats, AI systems interfacing with other hardware and software may face new security and privacy threats that have never existed before [57]. Malicious use and exploitation may also threaten the integrity of AI systems. Similar to other AI systems, health care AI systems, especially generative AI systems, are susceptible to code extraction and information extraction (eg, black-box, gray-box, and white-box attacks), leading to security and privacy breaches [57]. The excessive use of prompts may reveal copyright-protective data, proprietary research findings (eg, chemical compounds of a new drug), and training models or algorithms.

Table 3 summarizes the previously discussed security and privacy threats associated with each category of generative AI systems throughout their life cycle in health care.

**Table 3:** Generative artificial intelligence (AI) in health care categories and security or privacy threats in model training or building and implementation phases.

Category	Model training and building phase			Implementation phase	
	Integrity threats	Availability threats	Integrity threats		Confidentiality threats
Medical diagnostics	Adversarial training and classification manipulation (eg, image classification manipulation)	Model performance deteriorating by feeding poisonous data	AI hallucination (eg, made-up diagnosis), misinformation or disinformation, and adversarial use exploitation		Data extraction from carefully crafted prompts and privacy attacks
Drug discovery	Adversarial training and classification manipulation	Model performance deteriorating by feeding poisonous data	AI hallucination (eg, made-up chemical compound or protein structures), misinformation or disinformation, and adversarial use exploitation		Data extraction from carefully crafted prompts and privacy attacks
Virtual health assistants	Adversarial training and classification manipulation	Model performance deteriorating by feeding poisonous data	AI hallucination (eg, made-up medical advice), misinformation or disinformation, and adversarial use exploitation		Data extraction from carefully crafted prompts and privacy attacks
Medical research	Adversarial training and classification manipulation	Model performance deteriorating by feeding poisonous data	AI hallucination (eg, made-up findings, hypothesis, and citations), misinformation or disinformation, and adversarial use exploitation		Data extraction from carefully crafted prompts and privacy attacks
Clinical decision support	Adversarial training and classification manipulation	Model performance deteriorating by feeding poisonous data	AI hallucination (eg, made-up conclusions, findings, and recommendations), misinformation or disinformation, and adversarial use exploitation		Data extraction from carefully crafted prompts and privacy attacks

Again, it should be noted that some of these threats are unique to generative AI systems, but many of the threats are prevalent in broader AI systems in health care and other fields.

## Recommendations

### Overview

As security and privacy threats exist in the life cycle of various generative AI systems in health care, from data collection through model building to clinical implementation, a systematic approach to safeguard them is critical. This section provides some recommendations on safeguards. In doing so, we rely on the National Institute of Standards and Technology Privacy Framework and the National Institute of Standards and Technology AI Risk Management Framework as well as the regulatory guidance discussed in the Literature Review section. It should be noted that although the security and privacy threats discussed in this study are significant and some are unique in the context of generative AI in health care, many are also common in other types of AI models and other AI application contexts. Hence, many of the recommendations we propose in the subsequent section can be applied to AI in non–health care contexts.

### Development Protocols of Risk Assessment for Generative AI in Health Care

AI risks, including those of generative AI in health care, can emerge in a variety of ways at any phase of an AI project. Health care organizations need to learn from managing risks for other technologies to develop risk assessment protocols for generative AI in health care, along with risk assessment metrics.

#### AI Risk Assessment Protocols

To systematically manage AI risks, health care organizations must develop risk assessment protocols that include risk assessment procedures and methodologies by following industrial standards and frameworks as well as best practices [63]. A total of 3 main risk assessment activities are involved in the protocol development: risk identification, risk prioritization, and risk controls. All 3 activities must be conducted throughout the life cycle of a generative AI system in health care.

In the data collection and processing phase, health care organizations can use several methods to identify, prioritize, and control AI risks. As discussed before, health care data are messy and tend to have organic biases (eg, a hospital specializes in serving a particular patient demographic, attending to gender-specific health requirements or offering dedicated care for rare diseases). When collecting data or using GANs to generate synthetic data, the health care field needs to be extremely diligent. One recommendation is to establish data collection or generation policies and procedures. The separation of clinical and nonclinical data is necessary, given the significantly different risks in these 2 types of data. Similarly, the establishment of the metrics and methods to check training data on biases for clinical and nonclinical data is also important. Data provenance and authentication metrics can be used to prevent collecting data from untrustworthy sources; detecting and filtering methods can be used to identify and filter poisoned data; and data standardization improves the quality of data collection [57]. As the frontline defense, these prevention mechanisms can prevent integrity and availability attacks during this phase. Nevertheless, regardless of the mechanisms, data collected from medical sources or generated by GANs should reflect the comprehensive overview of a medical domain and the complexity of the physical and digital dimensions in such a domain to prevent biases and test for risks.

In the model training and building phase, detecting and filtering are also important for identifying and removing adversary training examples. Robustness, generalizability, and other vulnerability tests (eg, black-box and white-box tests) can further prevent integrity and availability attacks and data breaches [88]. Input reconstruction is another mechanism to pinpoint sources of adversary training [89]. Modifying training processes and models as well as training methods may also help to control AI risks in this phase [57]. Given the complexity and variety of AI models in reasoning and learning, we suggest a taxonomy approach. For example, a deep learning model can carry significantly different risks than a probabilistic learning model. By building a taxonomy of AI models and their risks, researchers can systematically identify and control security and privacy risks based on the AI model.

In the model implementation phase, routine verification and validation are key to identifying and controlling AI risks [63]. The implementation contexts of generative AI also matter. In some cases, verification and validation are about not only factual accuracy but also communications and perceptions as well as cultures. A medical chatbot that was thoroughly tested in adult populations may not be very useful in teenage populations. Gesture and face recognition AI for medical diagnosis may need to be culturally sensitive to be useful. When generative AI is integrated and interacts with other systems, for example, to create multiagent systems or medical robotics (eg, companion robots), security tests along with social, philosophical, and ethical tests are a must.

#### AI Risk Assessment Metrics

Given the complexity of AI security and privacy risks, health care organizations should develop risk assessment metrics for each of the 3 phases of the life cycle of a generative AI project. The following subsections highlight some measures for AI risk assessment metrics.

##### Security Objectives

AI risk assessment metrics should include well-established security and privacy objectives such as confidentiality, integrity, availability, nonrepudiation, authentication, and privacy protection. In the data collection and processing phase, collection technologies should be evaluated regardless of software- or hardware-based collection to ensure that they meet the security and privacy objectives. The use of synthetic medical data should follow the same security and privacy objectives to ensure that such data capture the factual and scientific truth. In the model training and building phase, vulnerability tests should be conducted to identify known and unknown threats based on security objectives. For example, availability attacks such as denial of service can be used to flood conversational health AI applications to assess their resilience and availability before deployment, and integrity attacks with poisoned data can be used to test the stability of model performance and generalizability [57]. In the implementation phase, all security objectives should be routinely assessed.

##### Generative AI–Specific Metrics

#### AI Inscrutability

AI inscrutability refers to the lack of understandability of an AI model and its outcomes [63]. Although AI inscrutability is not directly related to security and privacy, it adds obfuscations to AI risk assessment to identify threats and vulnerabilities as well as biases owing to the lack of transparency and explainability in AI, especially in generative AI based on deep learning. Although we have identified AI inscrutability as a key metric for generative AI assessment, we acknowledge that the challenge of inscrutability is not unique to generative AI and has been a long-standing issue in the broader field of AI, particularly in health care. Various algorithms used in patient matching, diagnosis, and other proprietary applications often lack transparency because of their closed nature or intellectual property constraints. Therefore, many of them, even those that are not based on generative techniques, face similar scrutiny regarding their lack of transparency. Hence, the call for greater openness and explainability applies broadly across AI applications in health care, reflecting a growing demand for accountable and interpretable AI systems.

Nevertheless, the problem of inscrutability becomes pronounced in the context of generative AI because of its complex and often opaque decision-making processes, which can amplify the challenges already faced in health care AI. Generative AI models, especially when based on deep learning, can operate as “black boxes,” making it even more difficult for practitioners to understand how conclusions or recommendations are derived. This opacity is a critical concern in health care, where explainability and trust as well as accountability are paramount for clinical acceptance and ethical practice.

To address these concerns, there is a need for concerted efforts toward developing more interpretable AI models and regulatory frameworks that mandate transparency in AI applications, including those used in patient care. These efforts should be complemented by initiatives to educate health care professionals about the workings and limitations of AI tools, enabling them to make informed decisions while using these technologies in clinical settings. Therefore, although the inscrutability of generative AI presents specific challenges owing to the complexity and novelty of these models, it is a continuation of the broader issue of transparency in health care AI. Recognizing this, our discussion of AI inscrutability not only highlights the unique aspects of generative AI but also situates it within the ongoing discourse on the need for greater transparency and accountability in all AI applications in health care.

#### AI Trustworthiness

AI trustworthiness is defined as the degree to which stakeholders of an AI system have confidence in its various attributes [63,90]. Trust has been a significant factor in IT adoption. The fundamental argument is that if an IT system automatically runs behind the scenes to assist the work and decisions of human users, a trusting relationship must be established for users to interact with and rely on the system [91]. Nevertheless, trust is a complex concept and is built upon human users’ interaction and consequent assessment of the system from cognitive, emotional, and social dimensions [91-93]. Since the emergence of AI, AI trustworthiness has caught significant attention in research, given the foreseeable complexity of human-AI interaction. The rise of generative AI has stimulated more discussions on this topic. The current consensus is that AI trustworthiness itself is a complex measurement with multiple dimensions, such as reliability, resilience, accuracy, and completeness [63,90]. Many other AI metrics or factors, such as transparency, explainability, robustness, fairness, and user interactions or perceptions, can be the antecedents of AI trustworthiness. AI trustworthiness can also be context dependent. For example, explainability and interaction experience can be the determinants of the AI trustworthiness of a chatbot application on the patient portal, whereas reliability, accuracy, and completeness are significant factors in the AI trustworthiness of a radiology diagnosis AI for radiologists. Given the complexity of measuring AI trustworthiness, we recommend developing context-specific AI trustworthiness metrics. Similar to AI inscrutability, although AI trustworthiness is not a direct measure of security and privacy risks, it helps reduce the probability and magnitude of such risks throughout the life cycle of generative AI in health care. For instance, accuracy and reliability help to improve the integrity of an AI system.

#### AI Responsibility

AI responsibility is another key measure in AI risk assessment. Again, although this measure does not directly evaluate security and privacy risks, it endorses responsible AI practices that facilitate the discovery of the negative consequences and risks of AI, including the security and privacy risks of generative AI. Moreover, this measure is centered on the uniqueness of AI, especially generative AI, in “human centricity, social responsibility, and sustainability” [63]. In other words, AI responsibility is a multifaceted measure depending on many other metrics and factors such as the ethical framework (eg, biases, fairness, and transparency) and legal perspective (eg, accountability and traceability). This is also an emerging concept that is under development. The development and deployment of generative AI add complexity to this measure owing to its possible, unintended, but profound negative consequences and risks to human society. In health care, there is a legal ambiguity related to AI responsibility. Hospitals are still unclear about their legal liability when facing an AI incident. Despite such legal uncertainty, responsible AI use should be the baseline. We recommend that health care organizations use AI for consultation and assistance instead of replacement, given legal ambiguity and uncertainty, while intensively exploring generative AI from the perspectives of patient centricity and social responsibility and asking serious questions. For example, a generative drug discovery AI may find a new molecular formula for a biochemical weapon. How can we responsibly use such AI without crossing the line of no harm to human beings? Such a question leads to another key measure for AI risk assessment—AI harm.

#### AI Harm

AI harm can occur to individuals, organizations, and societies. For example, AI may cause physical harm to individual patients, damage a hospital’s reputation owing to AI incidents, and even endanger society if it is weaponized (eg, being used to disrupt the global drug manufacturing and supply chain). Hence, AI harm is a risk measure highly related to AI responsibility and trustworthiness. Developing trustworthy AI and following responsible AI practices can reduce or avoid AI harm.

It is worth mentioning that some of the metrics we proposed here pass some human characteristics into AI. A crucial philosophical distinction must be made regarding the attribution of human characteristics such as trustworthiness and responsibility to generative AI systems versus the health care organizations and technology partners developing these algorithms. Although metrics aim to make models appear more trustworthy and responsible in reality, trust emerges from human-centered institutional processes, and responsibility stems from human accountability. It may be challenging to humanize AI systems and transfer attributes such as trustworthiness to the algorithms themselves. Indicators of model transparency, reliability, or accuracy may engender confidence among stakeholders, but public trust fundamentally arises from the ethical data governance, risk communication, and oversight procedures instantiated by organizations. Without robust governance and review processes overseeing development, data practices, and risk monitoring, claims of AI trustworthiness lack substantiation. Similarly, although algorithmic outputs highlighting potential issues such as biases or errors increase awareness, this does not intrinsically amount to AI responsibility. True accountability involves diligent human investigation of problems that surface, enacting appropriate recourse, and continuous authority oversight. Metrics may aim for AI to appear more responsible, but responsibility mainly manifests in organizational commitment to discovering issues, working with experts to properly assess AI harms, and instituting robust redress processes with stakeholder input. Thus, trustworthiness and responsibility are contingent on extensive institutional support structures rather than innate model capabilities. Although progress indicators may serve as signals for these desired attributes, establishing genuine public trust and accountability in health care ultimately falls on the shoulders of health care administrators, innovators, and engaged communities, rather than solely on the algorithms themselves. Clarifying this distinction enables us to properly set expectations and delineate responsibilities as generative AI becomes increasingly prevalent in critical medical settings.

### Conclusions

Integrating generative AI systems into health care offers immense potential to transform medical diagnostics, research, treatment planning, and patient care. However, deploying these data-intensive technologies also introduces complex privacy and security challenges that must be proactively addressed to ensure the safe and effective use of these systems. Examining diverse applications of generative AI across medical domains (ie, medical diagnostics, drug discovery, virtual health assistants, medical research, and clinical decision support) helps this study uncover vulnerabilities and threats across the life cycle of these systems, from data collection to model development to clinical implementation. Although generative AI enables innovative use cases, adequate safeguards are needed to prevent breaches of PHI and to maintain public trust. Strategies such as developing AI risk assessment protocols; formulating specific metrics for generative AI such as inscrutability, trustworthiness, responsibility, and harm; and ongoing model monitoring can help mitigate risks. However, developing robust governance frameworks and updates to data privacy regulations are also required to oversee these rapidly evolving technologies. By analyzing the use cases, impacts, and risks of generative AI across diverse domains within health care, this study contributes to theoretical discussions surrounding AI ethics, security vulnerabilities, and data privacy regulations. Future research and development in generative AI systems should emphasize security and privacy to ensure the responsible and trustworthy use of these AI models in health care. Moreover, the security and privacy concerns highlighted in this analysis should serve as a call to action for both the AI community and health care organizations looking to integrate generative AI. Collaborative efforts between AI developers, health care providers, policy makers, and domain experts will be critical to unlocking the benefits of generative AI while also prioritizing ethics, accountability, and safety. By laying the groundwork to make security and privacy the central pillars of generative AI in medicine, stakeholders can work to ensure that these transformative technologies are harnessed responsibly for patients worldwide.

## Abbreviations

AI: artificial intelligence
CorGAN: Correlation-Capturing Convolutional Generative Adversarial Networks
EHR: electronic health record
EU: European Union
FL: federated learning
GAN: generative adversarial network
IoMT: Internet of Medical Things
LLM: large language model
medGAN: medical generative adversarial network
PHI: protected health information
